# Synchronized cardiac and respiratory sparsity for rapid free-breathing cardiac cine MRI

**DOI:** 10.1186/1532-429X-16-S1-W26

**Published:** 2014-01-16

**Authors:** Li Feng, Leon Axel, Jian Xu, Daniel K Sodickson, Ricardo Otazo

**Affiliations:** 1 Bernard and Irene Schwartz Center for Biomedical Imaging, New York University School of Medicine, New York, New York, USA; 2 Siemens Medical Solutions, New York, New York, USA

## Background

For patients with impaired breath-hold capacity or arrhythmias, free breathing real-time cine MRI is preferred at the expense of compromised spatiotemporal resolution. Compressed sensing (CS) has been used to achieve higher spatiotemporal resolutions in real-time cine MRI, but the superposition of respiratory and cardiac motion limits temporal sparsity. In this work, we propose a novel approach that sorts out cardiac and respiratory motion into separated but synchronized dimensions and performs a joint multicoil CS reconstruction with different sparsity constraints on cardiac and respiratory dimensions. Golden-angle radial sampling was employed for flexible data sorting. In arrhythmias cases, data are also sorted according to cardiac cycles with different length to reconstruct both "normal" and "ectopic" cycles.

## Methods

**Figure 1:**
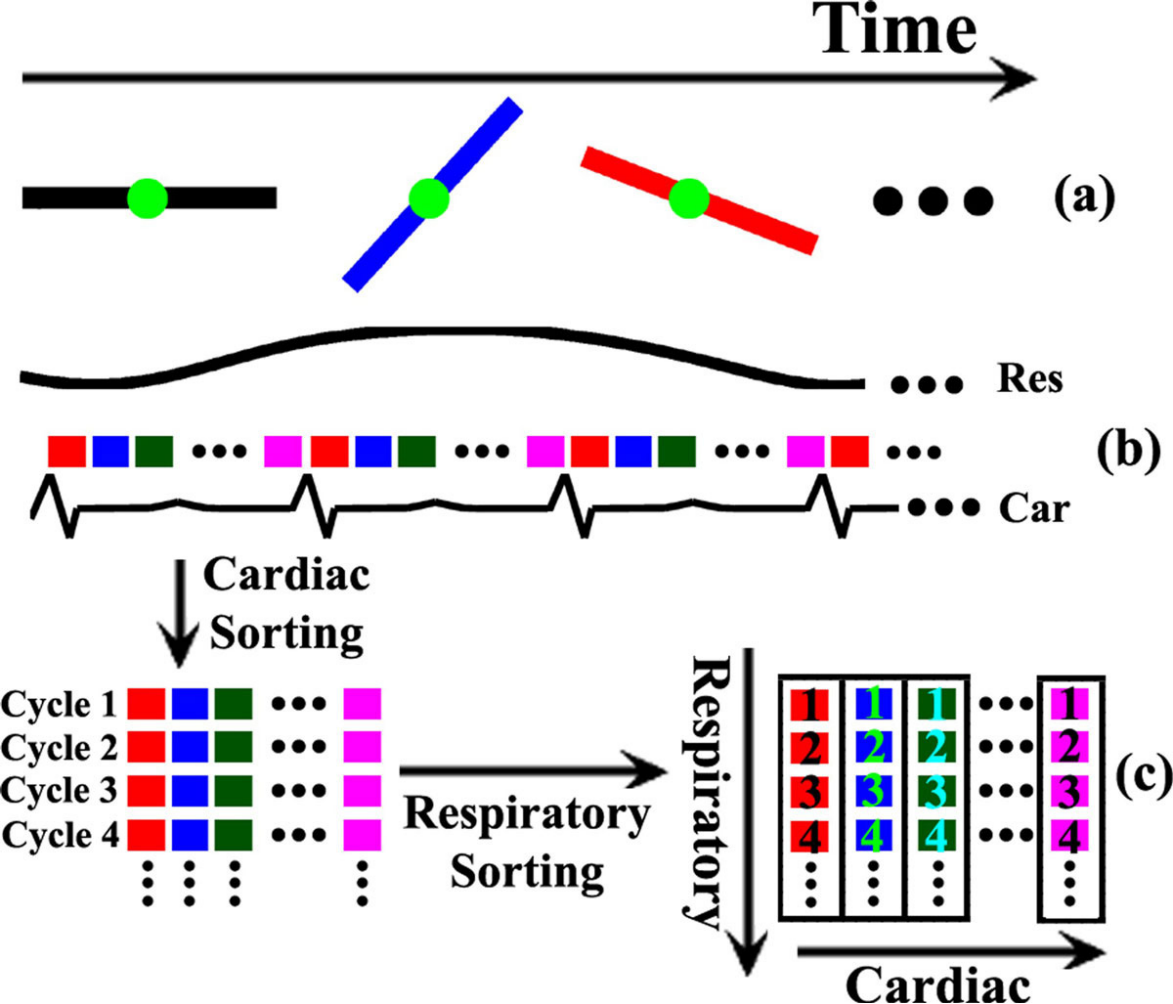
**(a) Continuous data acquisition**. (b) Cardiac (Car)/respiratory (Res) motion signals detected from the data itself. (c) Data are first sorted using the cardiac signal and then further sorted using the respiratory signal at each cardiac phase, as indicated by the black boxes.

Cardiac imaging was performed on one volunteer (male age = 27) and one patient (female age = 49) with Mobitz I arrhythmia during free breathing without external gating on a 1.5T MRI scanner (Avanto, Siemens). Data were continuously acquired for 15 s in a short axis plane using a 2D golden-angle radial b-SSFP sequence. Imaging parameters were: spatial resolution = 2 × 2 mm^2^, TR/TE = 2.8/1.4 ms, FA = 70° and slice thickness = 8 mm. Temporal evolution of the central k-space positions (green dots, Figure 1a) was used to estimate cardiac contraction and respiration from coil-elements close to the heart and diaphragm respectively (Figure 1b). Raw data were then sorted into an expanded dataset of images containing two dynamic dimensions, one for cardiac and the other for respiratory motion. As shown in Figure 1b, each colored rectangular block represents an individual cardiac phase from a short "snapshot" period (e.g. 13 adjacent spokes). Data were sorted first into a higher dimensional matrix using the cardiac motion signal (Figure 1c left) followed by a second sorting along the respiratory dimension from expiration to inspiration using the respiratory motion signal, performed within each black box shown in Figure 1c (right). For the arrhythmia patient, data from both "normal" and "ectopic" cycles were sorted separately according to the length of cardiac cycles so that reconstruction could be performed separately to produce both "normal" and "ectopic" cycles. CS reconstruction was performed with two total variation constraints along cardiac and respiratory dimensions. The results were also compared to Cartesian breath-hold approach using retrospective ECG-gating.

## Results

**Figure 2:**
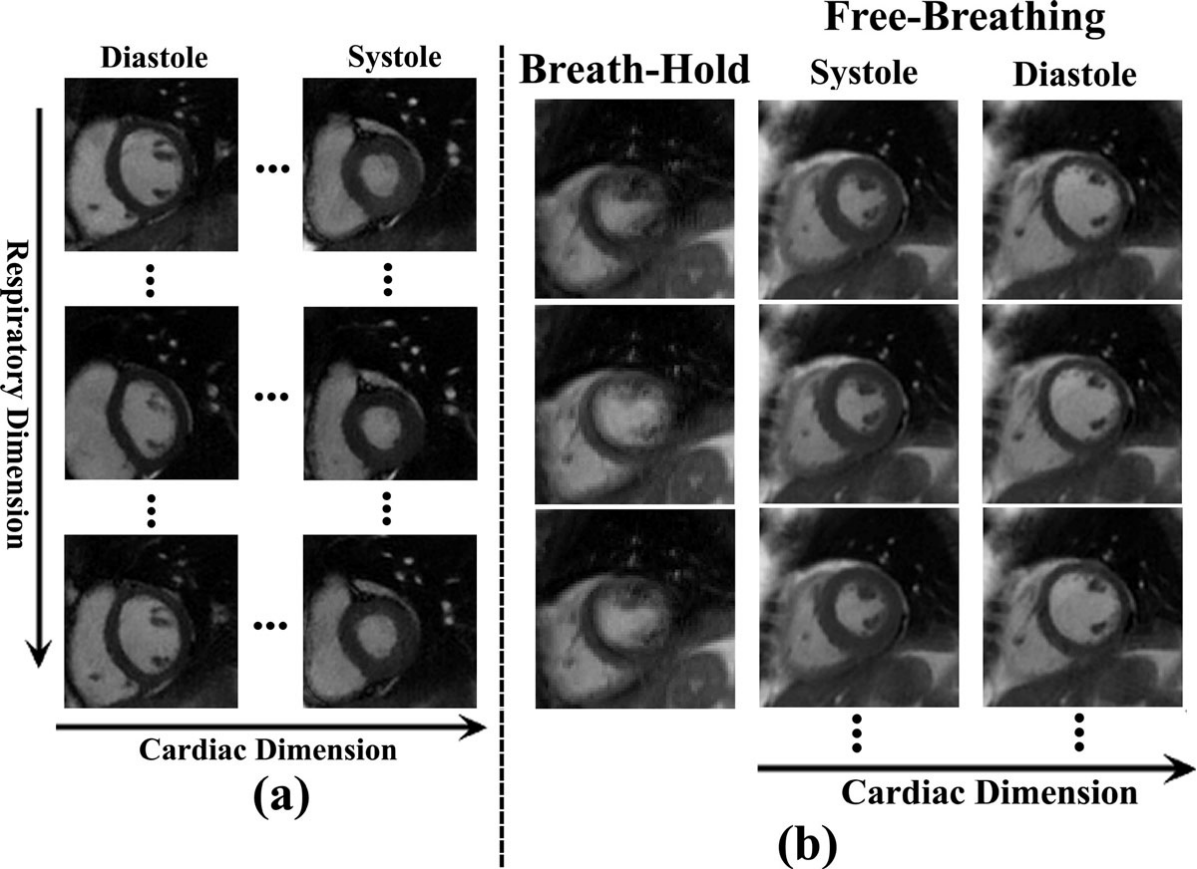
**(a) Cardiac cine imaging with cardio-respiratory synchronization in the volunteer**. Additional functional information can be obtained by evaluating the movement of myocardial wall along the respiratory motion in a given cardiac phase. (b) Clinical breath-held cardiac cine imaging using a Cartesian trajectory and retrospective ECG-gating in a patient with arrhythmia and corresponding free-breathing cardiac cine imaging with cardio-respiratory synchronization.

Figure 2a shows the different cardiac phases and respiratory states on the volunteer. Figure 2b compares the clinical breath-hold approach with the proposed method on the patient. Superior image quality is achieved even in the presence of arrhythmia.

## Conclusions

Separating cardiac and respiratory motion improved the sparsity of representation and thus the acceleration capability for CS. Additional functional information can be obtained by evaluating the movement of myocardial wall along the respiratory motion in a given cardiac phase.

